# The Norwich Neophyte

**Published:** 1891-05-16

**Authors:** 


### Passing Topics.

### THE NORWICH NEOPHYTE.

No one will doubt the truth of an observation recently made by Professor Case, of Oxford, in a letter to the *Times*, to the effect that considerable courage is requisite nowadays to deny a woman's right to do anything she has a mind to do —no matter what it is. And it has been made evident in divers cases that the more "pronounced" the lady's achievement is and the more radically it strikes at the distinction of sex, or the more graphically it illustrates a release from the restraints of an old-fashioned propriety, the more strenuous the efforts of her sympathisers to demonstrate that she is well within her rights.

In the case of Miss Adam, of the Norfolk and Norwich Hospital, none of the usual questions arise. Her action is altogether dissociated from the ordinary issues. It is not with her, "what man dares do I dare," but rather "what never before me man or woman dared, I do," and no wonder the result is awaited in the neighbourhood with interest bordering upon excitement.

At this period of the world's history a question of religious disability is one to be deplored, and we think that Miss Adam has been very ill-advised to allow it to be raised. If her change of religion is a matter for her own conscience and for that alone, the effect of that change upon her position as an officer of a public institution comes rightly within the purview of those under whom she works, and who are responsible for her appointment. It must have been perfectly well understood, rule or no rule, that she would not have been elected to her present post had she announced herself at the time of her candidature a Romanist, and therefore it seems to follow that the position ought not to be retained now that an essential qualification—her Protestantism—has been surrendered. The most straightforward, and certainly the most dignified course would have been to resign, and if there be reason to believe that the supporters of the hospital are not unwilling the post of Lady Superintendent shall continue to be held by Miss Adam, notwithstanding her change of creed, it is open to her to enter herself afresh as a candidate for the appointment. In this way, she would rightly put upon the governing body the responsibility of settling a question she can have no possible mandate to decide for herself.

We sincerely hope this is the view which will ultimately commend itself to Miss Adam, and that she will by adopting it relieve herself and the Committee from a most embarrassing position. We observe that the Duke of Norfolk, who, it is stated, had not previously attended a meeting of the Norfolk and Norwich Hospital, was present at the late gathering of governors and subscribers. We do not know what part, if any, the Duke took in the proceedings, but without direct evidence to the contrary, we shall do him the justice to believe that he was not there to support Miss Adam.

It would be one thing for the Duke to contend, as no doubt he would, that the hospital offices should be open to Roman Catholics; it would be another and quite a different thing to seek to uphold an official who boldly asserts possession to be rather more than nine points of the law, and whose sense of the conditions under which a responsible appointment is held is best expressed in the motto, *J'y suis; j'y reste.* There is a charming simplicity about this, but although the interests of the Institution seem the last thing thought of, they have their claims and the authorities must prove equal to the occasion. Agricola.

